# Smoking status and vascular risk factors as predictors of disability in AQP4-NMOSD and MOGAD

**DOI:** 10.1177/13524585251325069

**Published:** 2025-03-15

**Authors:** Fiona Chan, David Berhanu, Sara Samadzadeh, Anna Francis, Nasrin Asgari, Friedemann Paul, M Isabel Leite, Ruth Geraldes, Jacqueline Palace

**Affiliations:** Nuffield Department of Clinical Neurosciences, University of Oxford, Oxford, UK; Translational Neuroimmunology Group, Faculty of Medicine and Health, Sydney Medical School, University of Sydney, Sydney, NSW, Australia; Nuffield Department of Clinical Neurosciences, University of Oxford, Oxford, UK; Multiple Sclerosis Centre of Lisbon (CRI EM), ULS São José, Lisbon Clinical Academic Centre, Lisbon, Portugal; Neuroradiology Department, ULS São José, Lisbon Clinical Academic Centre, Lisbon, Portugal; Faculdade de Medicina da Universidade de Lisboa, Lisbon, Portugal; Nuffield Department of Clinical Neurosciences, University of Oxford, Oxford, UK; Institute of Regional Health Research, and Institute of Molecular Medicine, University of Southern Denmark, Odense, Denmark; Department of Neurology Næstved-Slagelse-Ringsted Hospitals, Slagelse, Denmark; Charité – Universitätsmedizin Berlin, corporate member of Freie Universität Berlin and Humboldt-Universität zu Berlin, Experimental and Clinical Research Center, Berlin, Germany; Nuffield Department of Clinical Neurosciences, University of Oxford, Oxford, UK; Institute of Regional Health Research, and Institute of Molecular Medicine, University of Southern Denmark, Odense, Denmark; Department of Neurology Næstved-Slagelse-Ringsted Hospitals, Slagelse, Denmark; Charité – Universitätsmedizin Berlin, corporate member of Freie Universität Berlin and Humboldt-Universität zu Berlin, Experimental and Clinical Research Center, Berlin, Germany; Nuffield Department of Clinical Neurosciences, University of Oxford, Oxford, UK; Department of Clinical Neurology, John Radcliffe Hospital, Oxford University Hospitals Trust, Oxford, UK; Nuffield Department of Clinical Neurosciences, University of Oxford, Oxford, UK; Department of Neurology, Frimley Health NHS Foundation Trust, Frimley, UK; Nuffield Department of Clinical Neurosciences, University of Oxford, Oxford, UK; Department of Clinical Neurology, John Radcliffe Hospital, Oxford University Hospitals Trust, Oxford, UK

**Keywords:** Smoking, comorbidities, vascular risk factors, aquaporin-4 antibody-positive neuromyelitis optica spectrum disorder, myelin oligodendrocyte glycoprotein antibody disease, disability

## Abstract

**Background:**

Smoking and vascular risk factors (VRFs) are reported to have adverse effects in multiple sclerosis but data are limited in aquaporin-4 antibody-positive neuromyelitis optica spectrum disorder (AQP4-NMOSD) and myelin oligodendrocyte glycoprotein antibody disease (MOGAD). This study aimed to measure their impact on disability.

**Methods:**

Smoking status was defined as never, past or current smokers and VRF comprised of ⩾1: hypertension, dyslipidemia, high body mass index or diabetes. Logistic regression models were fitted to predict their influence on recovery from *onset* attack and *first optic neuritis (ON)* attack.

**Results:**

A total of 442 patients were included. Current MOGAD smokers had a higher risk of disability from onset attack and first ON attack than never smokers (odds ratio (OR) 2.9, 95% confidence interval (CI) 1.3–6.9; OR 3.3, 95% CI 1.4–7.8). VRF in MOGAD was not predictive of disability. Current AQP4-NMOSD smokers and VRFs had a higher risk of residual disability from onset attacks (OR 7.5, 95% CI 2.1–27.7; OR 1.9, 95% CI 1.0–3.4). VRF was associated with higher risk of visual disability (OR 2.6, 95% CI 1.08–6.46) while smoking status was not.

**Conclusions:**

Current smoking status detrimentally influenced onset attack recovery in AQP4-NMOSD and MOGAD patients, including visual recovery in MOGAD. Non-smoking VRFs influenced clinical and visual outcomes in AQP4-NMOSD.

## Introduction

Aquaporin-4 antibody-positive neuromyelitis optica spectrum disorder (AQP4-NMOSD) and myelin oligodendrocyte glycoprotein antibody disease (MOGAD) are antibody-associated inflammatory diseases of the central nervous system with predominant involvement of the spinal cord and optic nerves. In contrast to multiple sclerosis (MS), relapses are the key contributor of disability. Identifying prognostic factors in relapse recovery is therefore important for disability prediction in AQP4-NMOSD and MOGAD. Modifiable factors such as vascular risk factors (VRFs) and smoking are globally prevalent and may impact clinical outcomes in these disorders.

VRFs, including smoking, have been associated with increased disability and reduced quality of life in MS.^1–3^ Smokers have been shown to have poor recovery from relapses in AQP4-NMOSD^4^ and increased risk of both magnetic resonance imaging (MRI) lesion persistence and poor clinical recovery in a combined cohort of AQP4-NMOSD and MOGAD patients.^5^ There are limited studies focusing on the prevalence of VRF^6^ and its correlation with neurological disability in AQP4-NMOSD.^7,8^ Dedicated VRF studies in MOGAD cohorts are absent.

Visual outcomes are pertinent in AQP4-NMOSD and MOGAD^9–11^ but the expanded disability status scale (EDSS) does not accurately represent visual disability.^12^ Investigating the additive effects of smoking and VRFs on neuro-axonal damage to visual pathways after an episode of optic neuritis (ON) has not been studied before and may add to our understanding of the pathophysiology in these disease processes.

The aim of this study was to examine the impact of VRF and smoking on recovery from the onset attack in AQP4-NMOSD and MOGAD with a further focus on visual disability and optical coherence tomography (OCT) parameters, in a large national referral cohort.

## Methods

### Study design and participants

There are two national commissioned services for NMOSD/MOGAD in the United Kingdom which provides specialised services support to patients with AQP4-NMOSD^13^ and MOGAD.^14^ This study was performed by the national NMOSD/MOGAD commissioned service in Oxford, UK which is led by two neurology consultants (IL and JP) with expertise in NMOSD/MOGAD, thus allowing consistency in serial clinical examination and therapeutic management. Patients with AQP4-NMOSD^13^ and MOGAD^14^ seen from inception to November 2023 were eligible for inclusion if they: were aged ⩾16 years, had available smoking and VRF information, had clinical data on recovery from the onset attack and had documentation of visual acuity (VA) at least 6 months from first attack of ON. Smoking and VRFs were captured with standardised prospectively collected patient-filled questionnaires, and clinic visit height and weight measurements. This was a retrospective analysis of prospectively collected data, and consent was obtained under ethics reference: 21/SC/0353.

### Clinical definitions and outcomes

Smoking status was defined as ‘never smokers’, ‘past smokers’ and ‘current smokers’. Non-smoking VRF included: hypertension, type 2 diabetes, dyslipidemia and raised body mass index (BMI) >25 kg/m^2^. Patients were classified as ‘without VRF’ or ‘with VRF’ if they had at least one of these VRFs regardless of their smoking status.

The primary outcomes were (a) clinical recovery from the *onset* attack according to smoking status or VRF status and (b) visual recovery from the *first* ON attack according to smoking or VRF status.

Clinical recovery from relapse was scored prospectively on the database by the treating NMO consultants (IL or JP) as ‘complete recovery’ (large improvement with independent function or full recovery to baseline function) or ‘residual disability’ (patients who did not meet above definition for complete recovery) at least 6 months from the onset attack.

‘Poor visual recovery’ was defined as visual disability with a VA worse than LogMAR 0.1 (Snellen 6/7.5) ⩾6 months after the first attack of ON. In those with simultaneous bilateral ON, the worse eye was included in the analysis. Patients with any pre-existing ophthalmological pathology leading to poor VA or VRF-related ocular pathology were excluded.

### Optical coherence tomography

A small subgroup analysis was performed on those who had OCT in Oxford at least 6 months from first episode of ON, and patients with recurrent episodes of ON were excluded. Details of OCT parameters and selection methods^15–17^ are included in the Supplemental Material.

## Statistical analysis

Analysis between cohorts and exploratory groups was performed using Mann–Whitney *U* test, Kruskal–Wallis test, Fisher’s exact test and chi-square (χ^2^) tests as appropriate. Multivariable logistic regression modelling was used and included age and sex as covariates if *p* < 0.1 on univariate analysis. The multivariable logistic regression model for the AQP4-NMOSD cohort was fitted with relapse treatment data in a separate model as there was a difference in treatment rates for current smokers. Clinical recovery (as categorical outcomes) was the dependent variable and smoking and VRF status were independent variables. Results were expressed as the odds ratio (OR) of complete clinical recovery compared to residual disability, with 95% confidence intervals (CIs). Statistical significance was set at *p* < 0.05.

All analyses were performed using Stata version 16.1 (StataCorp 2019, Stata Statistical Software: Release 16, College Station, TX: StataCorp LLC) and R studio (R Project for Statistical Computing, Version 4.4.1 ‘lme’ package).

## Results

A total of 442 adult patients (236 MOGAD, 206 AQP4-NMOSD) were included in the study (Supplemental Figure 1, excluded participants are detailed in Supplemental Table 1). The MOGAD cohort had a younger median age of onset of 35 years compared to the AQP4-NMOSD cohort with median onset age of 47 years (*p* < 0.001). The majority of the MOGAD cohort (70%) experienced complete recovery. In contrast, only 41% of the AQP4-NMOSD cohort experienced complete recovery. The maximum severity of disability in the MOGAD cohort was EDSS 8 and EDSS 9 in the AQP4-NMOSD cohort. The maximum severity of visual disability in both cohorts was no perception of light. Table 1 shows the demographic and clinical details according to smoking status and VRF categories and demonstrates there was no significant association between being in the VRF category and in a smoking category.

**Table 1.** Demographic and clinical characteristics.

	MOGAD					AQP4				
	Total	Never smokers	Past smokers	Current smokers	*p*-value	Total	Never smokers	Past smokers	Current smokers	*p*-value
	*n* = 236	*n* = 146	*n* = 58	*n* = 32		*n* = 206	*n* = 155	*n* = 30	*n* = 21	
Age at onset (years) median (range)	35 (28–47)	34 (28–48)	35 (29–46)	36 (28–48)	0.98	47 (35–60)	46 (34–60)	55 (46–62)	42 (30–56)	0.04
Female *n* (%)	141 (60%)	92 (63%)	32 (55%)	17 (53%)	0.42	172 (84%)	132 (86%)	24 (80%)	16 (76%)	0.38
Without VRF *n* (%) With VRF *n* (%) - Hypertension, *n* (%) - Dyslipidemia, *n* (%) - Type 2 diabetes, *n* (%) - Raised BMI, *n* (%)	156 (66%) 80 (34%) 26 (11%) 7 (3%) 15 (6%) 56 (24%)	97 (66%) 49 (34%) 12 (9%) 6 (4%) 12 (8%) 34 (23%)	41 (71%) 17 (29%) 8 (17%) 1 (2%) 2 (3%) 11 (19%)	18 (56%) 14 (44%) 6 (19%) 0 (0%) 1 (3%) 11 (34%)	0.38	110 (53%) 96 (47%) 37 (18%) 11 (5%) 45 (22%) 39 (19%)	81 (52%) 74 (48%) 31 (20%) 10 (6%) 35 (23%) 27 (17%)	15 (50%) 15 (50%) 4 (13%) 1 (3%) 5 (17%) 10 (33%)	14 (67%) 7 (33%) 2 (10%) 0 (0%) 5 (24%) 2 (10%)	0.42
Acute treatment Treatment received, *n* (%) No treatment received, *n* (%) Data not available	188 (79.6%) 47 (20%) 1 (0.4%)	117 (79.4%) 28 (19%) 1 (0.6%)	46 (79%) 12 (21%) 0 (0%)	25 (78%) 7 (22%) 0 (0%)	0.94	138 (67%) 33 (16%) 35 (17%)	102 (66%) 23 (15%) 30 (19%)	24 (80%) 4 (13%) 2 (6%)	12 (57%) 6 (29%) 3 (14%)	0.25

MOGAD: myelin oligodendrocyte glycoprotein antibody-associated disease; AQP4-NMOSD: aquaporin-4 antibody-positive neuromyelitis optica spectrum disorder. Data presented as median (range) or *n* (%). *p*-Values are from Mann–Whitney *U*, Kruskal–Wallis test, Fisher’s exact test and chi-square test as appropriate.

### The effect of smoking and VRF on clinical recovery from onset attack

In the univariate analysis, age was significant for both MOGAD and AQP4-NMOSD and so was added as an independent continuous variable to the multivariable logistic regression model along with smoking status (comparator never smoker) and VRF to predict clinical recovery.

#### MOGAD cohort

**Figure 1.**
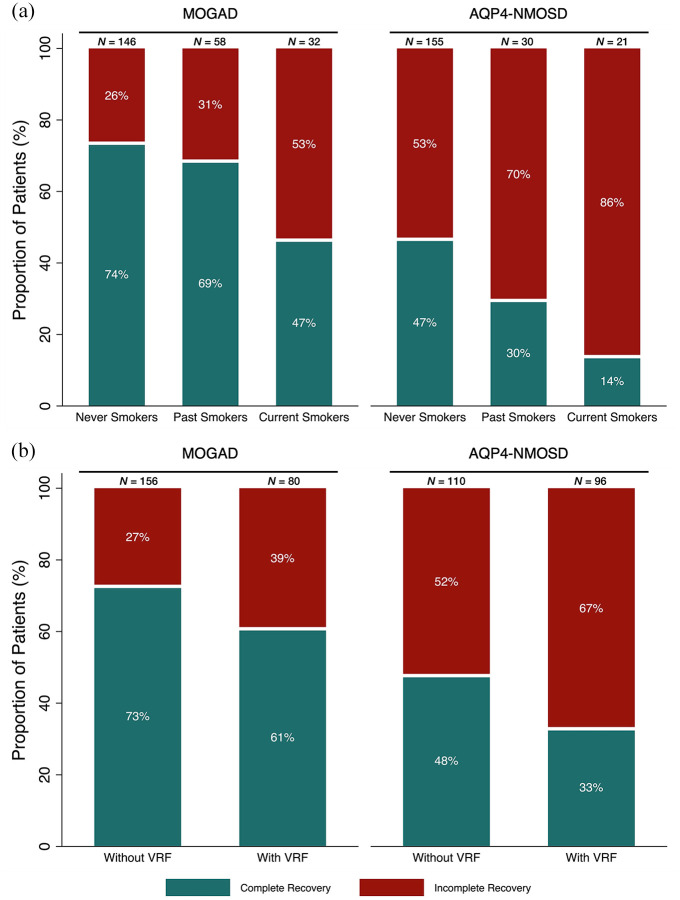
Percentage complete recovery (green) or residual disability/incomplete recovery (red) from onset attack in MOGAD and AQP4-NMOSD cohorts. (a) Dependent on smoking status. (b) Dependent on VRF status. MOGAD: myelin oligodendrocyte glycoprotein antibody-associated disease; AQP4-NMOSD: aquaporin-4 antibody-positive neuromyelitis optica spectrum disorder; VRF: vascular risk factor.

The majority of never smokers (74%) and past smokers (69%) fully recovered from their onset attack compared to less than half (47%) of current smokers (χ^2^ test = 9.0, *p* = 0.011, Figure 1a). Seventy-three percent of patients without VRF fully recovered from their onset attack compared to 61% of patients with VRF (χ^2^ test = 3.5, *p* = 0.063, Figure 1b).

In the multivariable logistic regression model, current smokers had higher risk of residual disability (OR 2.9, 95% CI 1.3–6.9) compared to never smokers. Past smokers did not have a significantly higher risk for residual disability compared to never smokers (OR 1.1, 95% CI 0.5–2.3). VRF was not associated with a higher risk of residual disability (OR 1.2, 95% CI 0.6–2.3, Table 2). Age remained marginally significant (OR 1.0, 95% CI 0.9–1.0).

**Table 2.** Multivariable model of the effect of smoking and VRF on recovery.

		MOGAD			AQP4-NMOSD		
		OR	95% CI	*p*-value	OR	95% CI	*p*-value
Recovery from onset attack	Past smokers	1.1	0.5–2.3	0.836	1.9	0.8–4.6	0.142
	Current smokers	2.9	1.3–6.9	0.011	7.5	2.1–27.7	0.002
	VRF	1.2	0.6–2.3	0.629	1.9	1.0–3.4	0.041
	Age	1.0	0.9–1.0	0.057	1.0	1.0–1.1	0.006
Recovery from first ON attack	Past smokers	1.2	0.5–2.7	0.659	2.3	0.7–8.2	0.186
	Current smokers	3.3	1.4–7.8	0.008	2.6	0.6–10.6	0.186
	VRF	1.7	0.8–3.3	0.142	2.6	1.1–6.5	0.032

MOGAD: myelin oligodendrocyte glycoprotein antibody-associated disease; AQP4-NMOSD: aquaporin-4 antibody-positive neuromyelitis optica spectrum disorder; VRF: vascular risk factors; ON: optic neuritis. The OR column represents odds ratio of residual disability from onset attack and visual disability from first optic neuritis attack.

#### AQP4-NMOSD cohort

Nearly, half (47%) of never smokers fully recovered from their onset attack compared to 30% of past smokers and only 14% of current smokers (χ^2^ test = 10.1, *p* = 0.007, Figure 1a). Forty-eight percent of patients without VRF fully recovered from the onset attack compared to 33% of patients with VRF (χ^2^ = 4.7, *p* = 0.031, Figure 1b).

In the multivariable logistic regression model, a higher risk of residual disability was found in current smokers (OR 7.5, 95% CI 2.1–27.7) and patients with VRF (OR 1.9, 95% CI 1.0–3.4) compared to never smokers and patients without VRF, respectively. Past smokers did not have a higher risk for residual disability than never smokers (OR 1.9, 95% CI 0.8–4.6, Table 2). Age remained significant (OR 1.0, 95% CI 1.0–1.1)

As less AQP4-NMOSD current smokers were treated for their onset attack in our cohort, we repeated the analysis with the subgroup in whom we had available relapse treatment data. This strengthened the smoking effect (OR 15.4, 95% CI 2.92–81.5) compared to never smokers, as acutely treated patients may have been those with more severe attacks.

### Effect of smoking and VRF on visual disability

A total of 181 MOGAD patients and 95 AQP4-NMOSD patients with clinical details of visual recovery from their first ON attacks were included. Age was not a significant predictive factor for recovery on univariate analysis for either disease group.

#### MOGAD cohort

**Figure 2.**
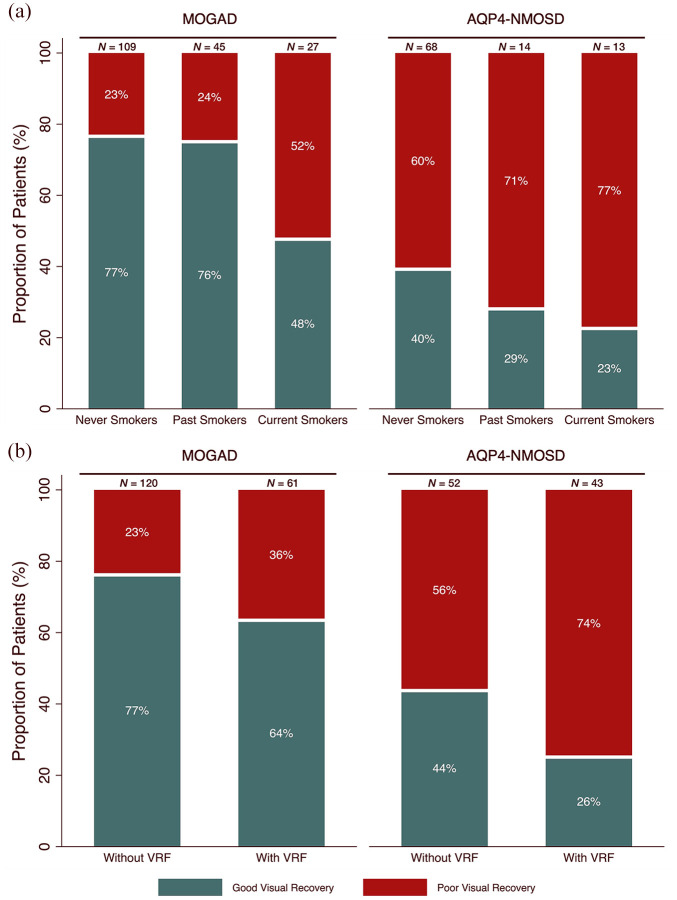
Percentage complete visual recovery (green) or poor visual recovery (red) from first ON attack in MOGAD and AQP4-NMOSD cohorts. (a) Dependent on smoking status. (b) Dependent on VRF status. ON: optic neuritis; MOGAD: myelin oligodendrocyte glycoprotein antibody-associated disease; AQP4-NMOSD: aquaporin-4 antibody-positive neuromyelitis optica spectrum disorder; VRF: vascular risk factor.

The majority of never smokers (77%) and past smokers (76%) experienced good visual recovery after first ON but less than half (48%) of current smokers had good visual recovery (χ^2^ test = 9.3, *p* = 0.009, Figure 2a).

A total of 77% of patients without VRF had good visual recovery compared to 64% of patients with VRF (χ^2^ test = 3.3, *p* = 0.070, Figure 2b).

In the multivariable logistic regression model, current smokers had an increased risk of visual disability (OR 3.3, 95% CI 1.4–7.8) compared to never smokers. In contrast, past smokers did not have an increased risk (OR 1.2, 95% CI 0.5–2.7) compared to never smokers. VRF was not a risk factor (OR 1.7, 95% CI 0.8–3.3) for visual disability.

#### AQP4-NMOSD cohort

Overall, a high proportion of patients had poor visual recovery, but a greater proportion of never smokers achieved good visual recovery (40%) compared to 29% of past smokers and 23% of current smokers (χ^2^ test = 1.7, *p* = 0.431, Figure 2a).

Forty-four percent of patients without VRF had good visual recovery compared to 26% with VRF (χ^2^ test = 3.6, *p* = 0.059, Figure 2b).

In the multivariable logistic regression model, VRF was significantly associated with worse visual recovery (OR 2.6, 95% CI 1.1–6.5, Figure 2b). Current smoking status and past smoking status were not significant risk factors for visual disability compared to never smokers (current smokers: OR 2.6, 95% CI 0.6–10.6; past smokers: OR 2.3, 95% CI 0.7–8.2, Table 2).

### OCT subgroup analysis

No significant effect of smoking status or VRF status on peripapillary retinal nerve fibre layer (p-RNFL) thickness, ganglion cell-inner plexiform layer (GCIPL) volume or total macular volume (TMV) ⩾6 months after first ON attack was found in MOGAD nor in AQP4-NMOSD.

Detailed results can be found in “Supplementary section: OCT subgroup analysis” section in the Supplemental Material, and number of eyes included across smoking and VRF cohorts are represented in Supplemental Tables 2 and 3 and Supplemental Figures 2 and 3.

## Discussion

Our study demonstrates detrimental effects of smoking and other VRF in MOGAD and AQP4-NMOSD. The impact of smoking status on ON and visual disability was investigated herein for the first time.

This study showed a higher proportion of MOGAD or AQP4-NMOSD patients had residual disability from their onset attack or their first ON attack if they were either current smokers or had at least one non-smoking VRF. In the multivariable regression analysis, only current smoking status was a significant risk factor in MOGAD for the onset attack and visual recovery from first ON. Both current smoking status and VRF were significant risk factors in AQP4-NMOSD recovery from onset attacks, but only VRFs were significant for visual recovery from first ON attack in AQP4-NMOSD. OCT was not sensitive to detect the effect of VRF and smoking across sub-groups.

The deleterious effect of smoking on MOGAD and AQP4-NMOSD clinical outcomes has been documented with ever smokers showing poorer recovery from relapses compared to never smokers in AQP4-NMOSD.^4^ Of note, smoking did not impact relapse rate nor time to relapse and no significant effect was noted in a smaller MOGAD cohort.^4^ The study however was underpowered to distinguish the different effects in ‘current smokers’ versus ‘past smokers’. In a study with a combined cohort of MOGAD and AQP4-NMOSD patients, smokers were also more likely to show lesion persistence on MRI after an acute attack which was correlated with poor clinical recovery.^5^ However, again the numbers were small and so the effects in the individual diseases could not be assessed. Our study included a much larger number of patients and was able to show that ‘current’ smokers had a poorer recovery from onset attack compared to ‘never’ smokers in both AQP4-NMOSD and MOGAD. It is noteworthy that past smokers did not appear to be at greater risk of poor recovery, highlighting the benefit of smoking cessation strategies. Although current smokers with AQP4-NMOSD had a higher risk of poor visual recovery, we were not able to detect a significant effect of smoking on visual outcomes in our AQP4-NMOSD cohort. This may be due to underpowering, as fewer AQP4-NMOSD patients had ON compared to the MOGAD cohort.

Limited studies of VRF have identified hypertension, diabetes and hyperlipidemia as prevalent comorbidities in AQP4-NMOSD.^6,7^ Cai et al.^7^ examined the impact of non-immune comorbidities on nadir vision and motor attacks in NMOSD. They observed greater ‘nadir’ of attacks in patients with any non-immune comorbidities and a higher risk of neurological disability at last follow up (defined as EDSS >6) in diabetes but not hypertension. Recovery from attack was not studied. Cho et al.^8^ correlated lipid profiles of 39 AQP4-NMOSD patients with neurological disability and found higher triglycerides, adjusted for steroid use, was associated with worse EDSS. We have not identified studies examining the impact of VRF on the MOGAD cohort or on visual disability outcomes in either AQP4-NMOSD or MOGAD. One study found higher BMI was significantly associated with a diagnosis of MOGAD in first episode of acute ON compared to AQP4-NMOSD or MS although the predictive value of BMI in MOGAD ON outcomes was not explored.^18^

Even though a higher proportion of current smokers and VRF cohort had poor recovery from onset attack and first ON attack in both disease groups, the VRF cohort did not reach statistical significance in MOGAD nor did current smoking status for visual recovery in AQP4-NMOSD. There could be various explanations for this. First, there is potential underpowering of the current study, especially within the AQP4-NMOSD cohort, as very few patients with ON were smokers and the visual recovery generally for all patients is poor. Repeating the study with larger numbers may reveal the effects of smoking and VRFs to be significant in clinical and visual outcomes in both diseases. In addition, MOGAD patients were younger and thus it is possible that ‘age-related’ VRF effects^19^ may not have been present for long enough to show an effect. Smoking however usually commences in the teens and as current smokers, rather than a past smoking history appears to be the major risk factor, the smoking effect appears to be more immediate and potentially reversible. Finally, these disorders have distinctive underlying disease mechanisms. AQP4-NMOSD is a primary astrocytopathy, where antibodies target AQP4 at end feet of astrocytes present on central nervous system capillary vessels,^20^ whereas in MOGAD, antibodies target the myelin oligodendrocyte glycoprotein (MOG) which is uniquely expressed on myelin and oligodendrocytes.^21^ In addition, AQP4 is expressed in retinal Müller cells and astrocytes, which are located in the retinal nerve fibre layer^22^ while the retina is typically unmyelinated, and therefore would be devoid of MOG. These distinctive features may contribute to different interactions with smoking and VRF effects.

The harmful effects of smoking and VRF are attributed to diverse pathophysiology. Smoking-related damage to the vascular and central nervous system is attributed to the release of pro-inflammatory cytokines, gas-related (nitrous oxide, carbon monoxide, cyanide) and hypoxic damage^23^ and complement activation.^24^ This might explain the difference in the effect of ‘current’ versus ‘past’ smoking. Although inflammatory damage is present in vascular comorbidities, the associated vasculopathy in these conditions is a result of a complex interplay of various metabolic and pro-inflammatory factors such as atherosclerosis, endothelial dysfunction, structural remodelling and advanced glycation end products from hyperglycemia.^25^ Thus, there may be different pathological processes between smoking and VRF which contribute differently to clinical outcomes. The lack of association noted in our study between smoking status and VRF is interesting and may reflect the distinct characteristics that drive smoking initiation in youth, or that patients with VRF are advised against smoking more than those without.

We did not find any significant associations between smoking and VRF on OCT parameters. However, OCT is an extremely sensitive clinical tool in AQP4-NMOSD and MOGAD ON^26,27^ and thus it may not be a good tool to detect subtle difference between sub-groups. As visual impairment after ON in AQP4-NMOSD is common, abnormalities across all sub-groups are not unexpected, whereas although MOGAD patients experience better visual recovery, the OCT is particularly sensitive in this disease showing abnormalities even when the VA outcomes are good.^28^

We acknowledge limitations within this study. Despite the overall large cohort, ‘current’ smokers were small in numbers (*n* = 32 MOGAD and *n* = 21 AQP4-NMOSD). Although there was no clear difference in the baseline features between the included and excluded cohorts, we are unable to rule out undetectable selection bias. Unknown confounders that might influence the results are also a limitation that we could not adjust for. We did not have detailed information on smoking such as duration of smoking and number of packs/per year and the length of time patients have ceased smoking in the past smoking group. However, it would be difficult to account for all these variables. In addition, we were not powered to distinguish between the sub-types or severities of each VRF. However, these are rare diseases and despite the relatively small cohort, we were still able to demonstrate higher proportions of poor outcomes in smokers and those with VRFs even if statistical significance was not achieved for some analyses. Categorical classification of risk factors may overlook effects at higher severity levels, potentially missing important associations. However, this does not negate the positive associations that have been observed.

This is the first study to systematically examine the impact of smoking and VRF on clinical outcomes in a large cohort of AQP4-NMOSD and MOGAD patients separately and to study visual outcomes with prospectively collected data. This study supports a detrimental effect of smoking on a large cohort of MOGAD and AQP4-NMOSD patients and VRF effects in AQP4-NMOSD, with an additional focus on visual recovery outcomes. These findings suggest that active management of VRFs and smoking cessation may be important for optimising patient outcomes in MOGAD and AQP4-NMOSD.

## Supplemental Material

sj-docx-1-msj-10.1177_13524585251325069 – Supplemental material for Smoking status and vascular risk factors as predictors of disability in AQP4-NMOSD and MOGAD Supplemental material, sj-docx-1-msj-10.1177_13524585251325069 for Smoking status and vascular risk factors as predictors of disability in AQP4-NMOSD and MOGAD by Fiona Chan, David Berhanu, Sara Samadzadeh, Anna Francis, Nasrin Asgari, Friedemann Paul, M Isabel Leite, Ruth Geraldes and Jacqueline Palace in Multiple Sclerosis Journal
