# Supplementary material for: Smoking status and vascular risk factors as predictors of disability in AQP4-NMOSD and MOGAD
Source: Mult Scler. 2025 Mar 15;31(6):658–67. doi: 10.1177/13524585251325069 (PMC12092943; doi:10.1177/13524585251325069)
Supplement: sj-docx-1-msj-10.1177_13524585251325069 – Supplemental material for Smoking status and vascular risk factors as predictors of disability in AQP4-NMOSD and MOGAD [file sj-docx-1-msj-10.1177_13524585251325069.docx]

**
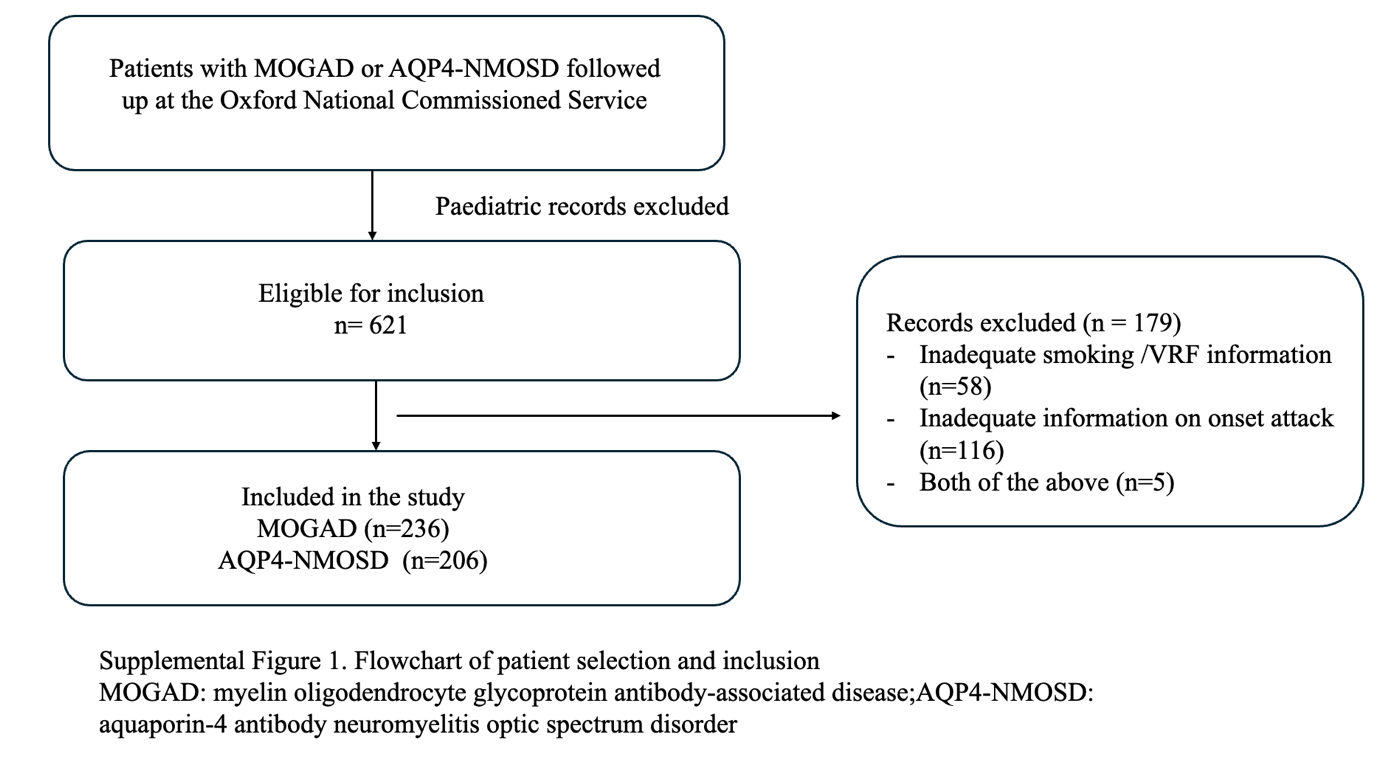
**

**
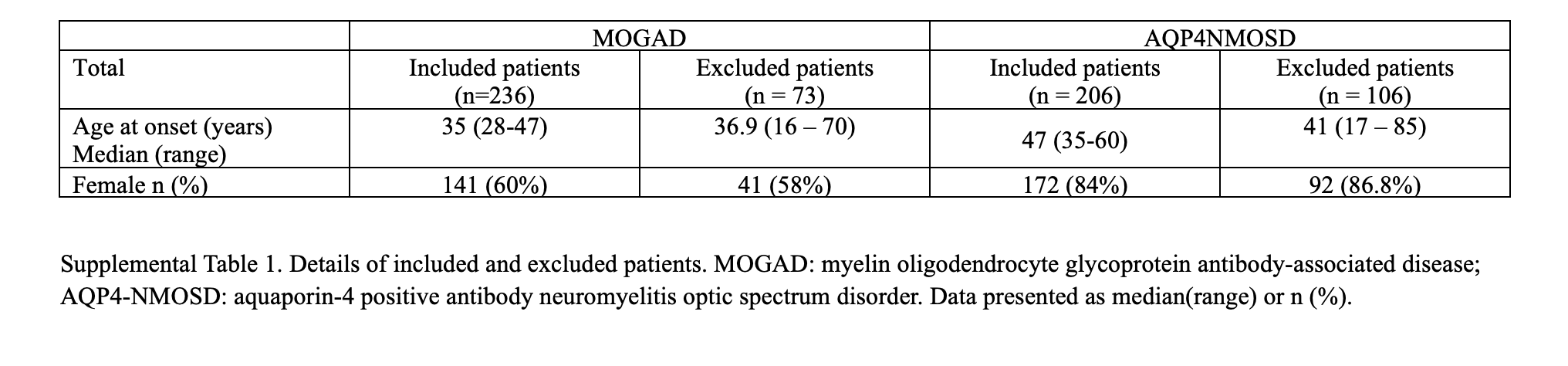
**

**Supplementary section: OCT subgroup analysis**

1. **Methods**

All OCT scans were performed in the same centre with Spectralis SD-OCT (Heidelberg Engineering, Heidelberg, Germany, software V.6.16.6_INT). OCT parameters studied were global peripapillary retinal nerve fibre layer (p-RNFL) thickness, combined ganglion cell and inner plexiform layer (GCIPL) volume and total macular volume (TMV). p-RNFL thickness was measured per device protocol, Spectralis peripapillary ring scan with a 12° or ~3.4 mm diameter around the optic nerve head. Total volume measurements were obtained by automatic segmentation of the macula with a grid diameter of 1, 3 and 6-mm centred on the fovea, as per the nine Early Treatment Diabetic Retinopathy Study (ETDRS) grid sectors. Individual OCT scans were only included if they exhibited signal strength greater than 20 dB, absence of artifact or retinal cystoid changes and performed in accordance with the OSCAR-IB criteria [1,2] and reported according to APOSTEL 2.0 recommendations [3].

1. **Statistical analysis**

Linear mixed-effects modeling was used to evaluate the impact of smoking status and other vascular risk factor on continuous OCT parameters. For the mixed-effects models, we tested assumptions of linearity, normality, and homoscedasticity of residuals, as well as the independence of random effects. To address variability in the data structure and relationships between random effects, we used an unstructured covariance matrix, which allowed the most flexibility in modelling correlations without imposing restrictive assumptions. The model included random effects to account for within-subject variability and fixed effects for the entire cohort analysis included age, as sex was not a significant factor. Regarding the exclusion of sex, we carefully evaluated its role in prior analyses of OCT results and in the current study. In both cases, including sex as a covariate did not significantly impact the OCT outcomes or improve model fit. Furthermore, the known gender imbalance in NMOSD and MOGAD cohorts, combined with its minimal influence on OCT results, led us to exclude sex to streamline the model and reduce the risk of overfitting. The unaffected eye was not used as a control because it was felt possible that the risk factors might influence the baseline OCT measures.

1. **Results**

In the MOGAD cohort, a total of 36 eyes were included for p-RNFL analysis and 34 eyes were included for GCIPL and TMV analysis. In the AQP4-NMOSD cohort, a total of 29 eyes were included for p-RNFL analysis and 25 eyes for GCIPL and TMV analysis. The median time from ON to OCT included in the MOGAD cohort was 1.5 years (6 months – 10.7 years) and 2.5 years in the AQP4-NMOSD cohort (6 months to 11.4 years). No significant effect of smoking status or VRF status on p-RNFL thickness, GCIPL volume or TMV ≥ 6 months after first ON attack was found in MOGAD nor AQP4-NMOSD using a linear mixed-effects modeling with age as a random factor.

| **Supplementary Table 2:** Impact of Smoking on OCT Parameters in AQP4-NMOSD and MOGAD Patients | | | | | | | |
| --- | --- | --- | --- | --- | --- | --- | --- |
|  | Never smoker  (No. of eyes) | Past smoker  (No. of eyes) | Active smoker  (No. of eyes) | Past Smoker vs Never Smoker: Estimate (SE, df, t Value, P Value) | Active Smoker vs Never Smoker: Estimate (SE, df, t Value, P Value) | R^2^_marg_ | R^2^_cond_ |
|  | pRNFL thickness | | | | | | |
|  | Thickness (μm, mean ± SD) | | |  | | | |
| AQP4-NMOSD with ON | 54.83 ± 14.01 (18) | 49.86 ± 11.78 (7) | 49.00 ± 16.45 (4) | -4.98 (6.31, 24, -1.04, 0.311) | -5.83 (7.96, 24, -0.73, 0.471) | 0.09 | 0.89 |
| MOGAD with ON | 73.2 ± 12.81 (20) | 67.18 ± 9.58 (11) | 63.00 ± 10.05 (5) | -6.02 (4.41, 31, -1.12, 0.271) | -10.2 (5.96, 31, -1.37, 0.181) | 0.16 | 0.90 |
|  | GCIPL volume | | | | | | |
|  | Volume (mm³, mean ± SD) | | |  | | | |
| AQP4-NMOSD with ON | 1.27 ± 0.34 (15) | 1.26 ± 0.28  (7) | 1.28 ± 0.38 (3) | -0.02 (0.15, 19, -0.26, 0.798) | 0.00 (0.21, 19, 0.00, 0.998) | 0.16 | 0.90 |
| MOGAD with ON | 1.59 ± 0.28 (20) | 1.64 ± 0.24 (10) | 1.39 ± 0.13 (4) | 0.05 (0.09, 28, 0.87, 0.390) | -0.2 (0.13, 28, -1.23, 0.228) | 0.22 | 0.90 |
|  | Total macular volume | | | | | | |
|  | Volume (mm³, mean ± SD) | | |  | | | |
| AQP4-NMOSD with ON | 7.34 ± 1.19 (15) | 6.90 ± 0.79  (7) | 7.29 ± 1.05 (3) | -0.44 (0.42, 20, -1.17, 0.257) | -0.05 (0.59, 20, -0.33, 0.745) | 0.34 | 0.92 |
| MOGAD with ON | 8.10 ± 0.67 (20) | 8.28 ± 0.52 (10) | 8.02 ± 0.47 (4) | 0.18 (0.24, 29, 1.01, 0.319) | -0.08 (0.34, 29, -0.04, 0.970) | 0.09 | 0.89 |
| Abbreviations: AQP4-NMOSD: Aquaporin-4 positive neuromyelitis optica spectrum disorder; pRNFL: Peripapillary retinal nerve fiber layer; GCIPL: Ganglion cell-inner plexiform layer; Total macular volume (TMV); SD: Standard deviation; ON: Optic neuritis; B: Estimate; SE: Standard errors; R^2^ marginal (R^2^ _marg_): Proportion of variance explained by the fixed factors alone; R^2^ conditional (R^2^ _cond_): Proportion of variance explained by both the fixed and random factors; All p-values > 0.05, indicating non-significance. | | | | | | | |

| **Supplementary Table 3:** Impact of Other Vascular Risk Factors on OCT Parameters in AQP4-NMOSD and MOGAD Patients | | | | | | | | |
| --- | --- | --- | --- | --- | --- | --- | --- | --- |
|  | At least one vascular risk factor  (No. of eyes) | No vascular risk factor  (No. of eyes) | Absolute difference  (μm, mean) | B | SE | P value | R^2^_marg_ | R^2^_cond_ |
|  | pRNFL thickness | | | | | | | |
|  | Thickness (μm, mean ± SD) | |  | | | | | |
| AQP4-NMOSD with ON | 47.83 ± 12.47 (12) | 56.35 ± 13.59 (17) | -8.52 | -10.08 | 5.21 | 0.064 | 0.16 | 0.90 |
| MOGAD with ON | 66.00 ± 9.70 (17) | 73.47 ± 12.85 (19) | -7.47 | -5.76 | 4.95 | 0.253 | 0.13 | 0.89 |
|  | GCIPL volume | | | | | | | |
|  | Volume (mm³, mean ± SD) | |  | | | | | |
| AQP4-NMOSD with ON | 1.20 ± 0.25 mm³ (11) | 1.33 ± 0.36 mm³ (14) | -0.12 | -0.16 | 0.13 | 0.210 | 0.22 | 0.90 |
| MOGAD with ON | 1.47 ± 0.26 mm³ (16) | 1.67 ± 0.22 mm³ (18) | -0.20 | -0.12 | 0.11 | 0.271 | 0.18 | 0.90 |
|  | Total macular volume | | | | | | | |
|  | Volume (mm³, mean ± SD) | |  | | | | | |
| AQP4-NMOSD with ON | 7.26 ± 0.77 mm³ (11) | 7.17 ± 1.27 mm³ (14) | +0.09 | -0.16 | 0.39 | 0.687 | 0.32 | 0.92 |
| MOGAD with ON | 7.95 ± 0.73 mm³ (16) | 8.31 ± 0.40 mm³ (18) | -0.35 | -0.26 | 0.27 | 0.346 | 0.09 | 0.89 |
| Abbreviations: AQP4-NMOSD: Aquaporin-4 positive neuromyelitis optica spectrum disorder; pRNFL: Peripapillary retinal nerve fibre layer; GCIPL: Ganglion cell-inner plexiform layer; Total macular volume (TMV); SD: Standard deviation; ON: Optic neuritis; B: Estimate; SE: Standard errors; R^2^ marginal (R^2^ _marg_): Proportion of variance explained by the fixed factors alone; R^2^ conditional (R^2^ _cond_): Proportion of variance explained by both the fixed and random factors; All p-values > 0.05, indicating non-significance. | | | | | | | | |


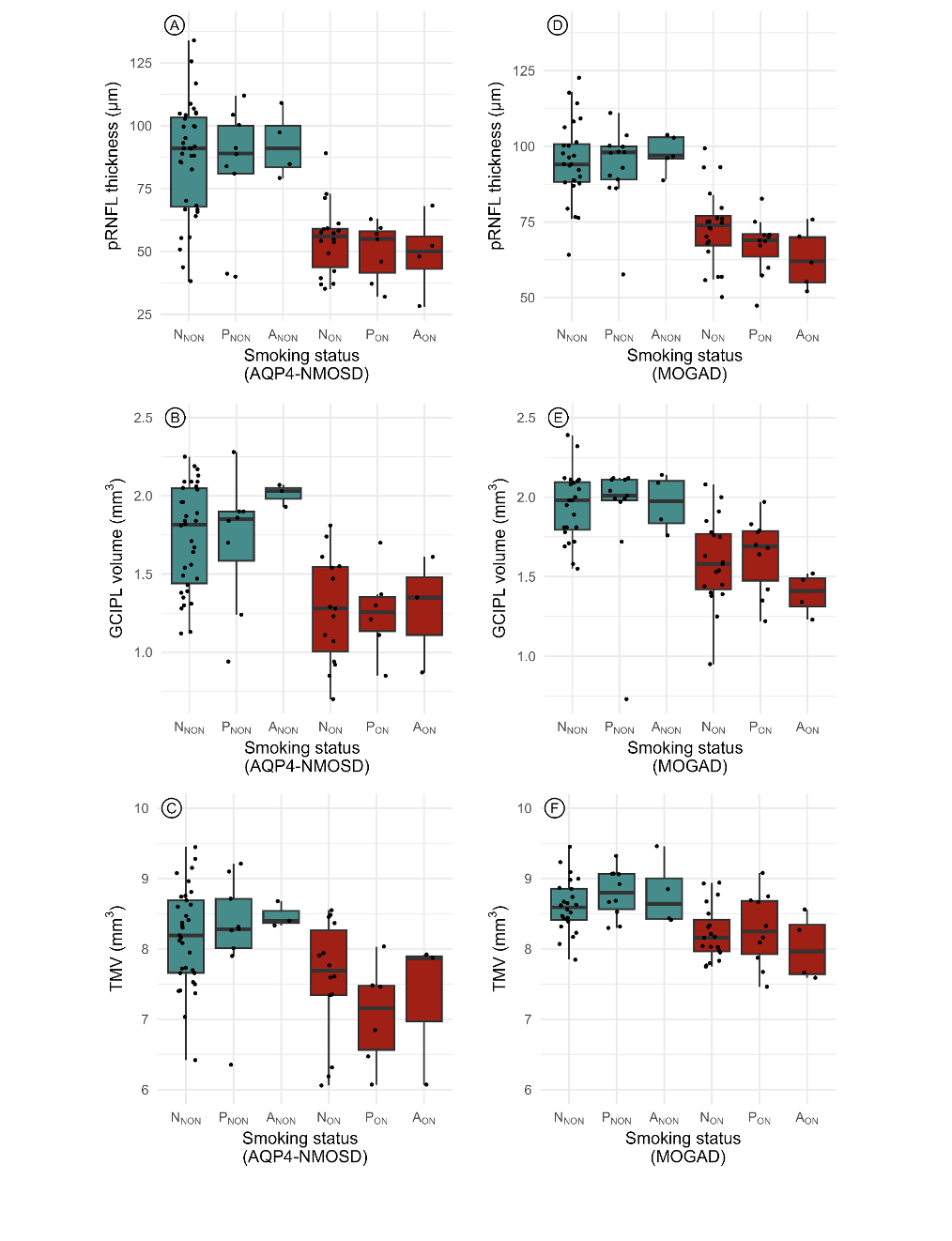


**Supplementary Figure 2:** Impact of smoking status on OCT Parameters in AQP4-NMOSD and MOGAD

(A) pRNFL thickness in AQP4-NMOSD patients by smoking status; (B) GCIPL volume in AQP4-NMOSD patients by smoking status; (C) TMV in AQP4-NMOSD patients by smoking status; (D) pRNFL thickness in MOGAD patients by smoking status; (E) GCIPL volume in MOGAD patients by smoking status; (F) TMV in MOGAD patients by smoking status. pRNFL: Peripapillary Retinal Nerve Fiber Layer; GCIPL: Ganglion Cell-Inner Plexiform Layer; TMV: Total Macular Volume; AQP4-NMOSD: Aquaporin-4 Positive Neuromyelitis Optica Spectrum Disorder; MOGAD: Myelin Oligodendrocyte Glycoprotein Antibody Disease; N: Never smoker; P: Past smoker; A: Active smoker; ON: Optic Neuritis; NNON: Never smoker without Optic Neuritis; PNON: Past smoker without Optic Neuritis; ANON: Active smoker without Optic Neuritis; NON: Never smoker with Optic Neuritis; PON: Past smoker with Optic Neuritis; AON: Active smoker with Optic Neuritis.


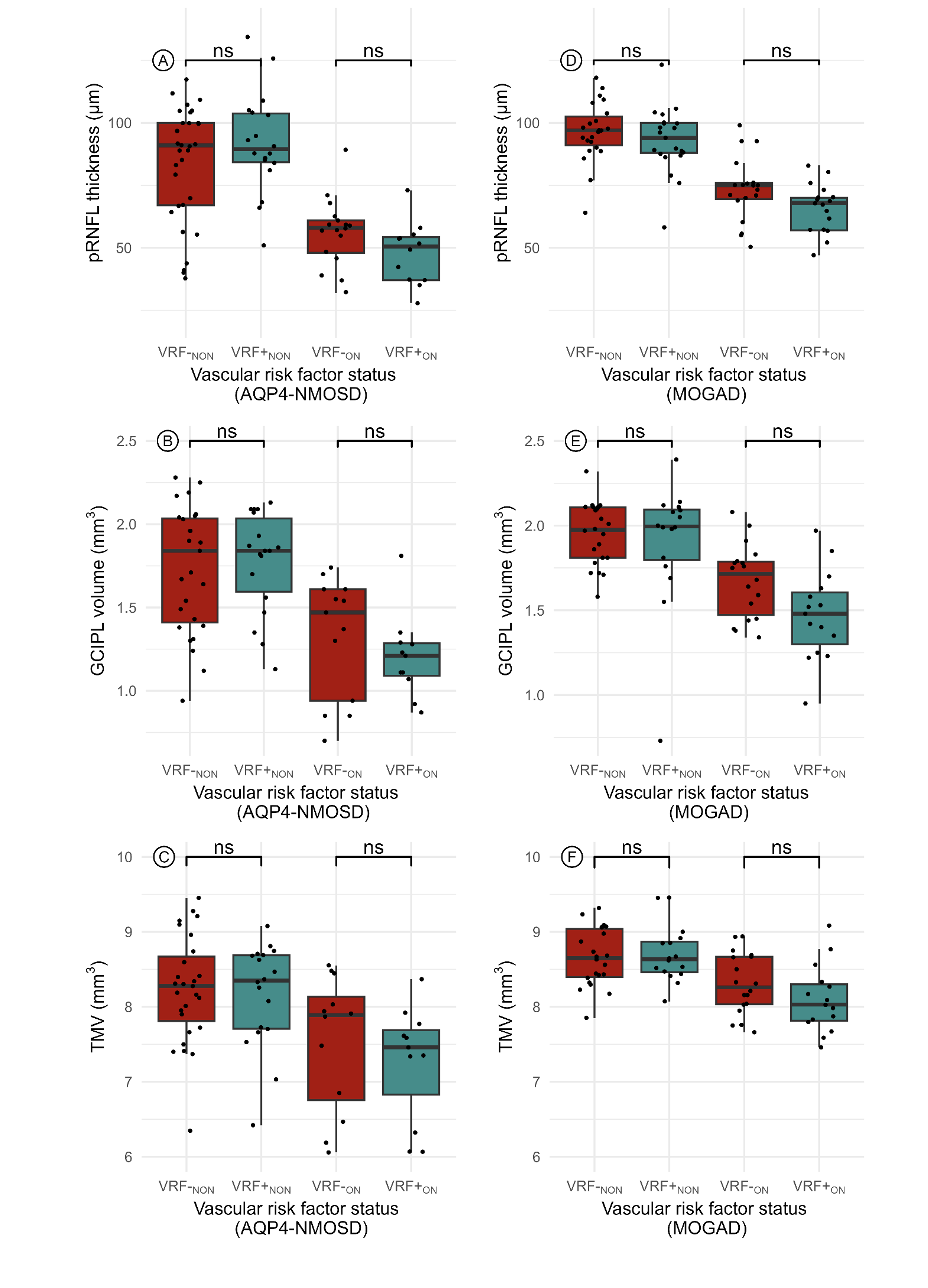


**Supplementary Figure 3:** Impact of Vascular Risk Factor status on OCT Parameters in AQP4-NMOSD and MOGAD (A) pRNFL thickness in AQP4-NMOSD patients; (B) GCIPL volume in AQP4-NMOSD patients; (C) TMV in AQP4-NMOSD patients; (D) pRNFL thickness in MOGAD patients; (E) GCIPL volume in MOGAD patients; (F) TMV in MOGAD patients.

pRNFL: Peripapillary Retinal Nerve Fiber Layer; GCIPL: Ganglion Cell-Inner Plexiform Layer; TMV: Total Macular Volume; AQP4-NMOSD: Aquaporin-4 Positive Neuromyelitis Optica Spectrum Disorder; MOGAD: Myelin Oligodendrocyte Glycoprotein Antibody Disease; VRF⁻NON: Vascular Risk Factor Negative without Optic Neuritis; VRF⁺NON: Vascular Risk Factor Positive without Optic Neuritis; VRF⁻ON: Vascular Risk Factor Negative with Optic Neuritis; VRF⁺ON: Vascular Risk Factor Positive with Optic Neuritis; ns: Not Significant

References

1. Tewarie P, Balk L, Costello F, et al. The OSCAR-IB consensus criteria for retinal OCT quality assessment. PLoS One 2012; 7: e34823. doi: 10.1371/journal.pone.0034823.

2. Schippling S, Balk LJ, Costello F, et al. Quality control for retinal OCT in multiple sclerosis: validation of the OSCAR-IB criteria. Mult Scler. 2015 Feb;21(2):163-70. doi: 10.1177/1352458514538110.

3. Aytulun A, Cruz-Herranz A, Aktas O, et al. APOSTEL 2.0 Recommendations for Reporting Quantitative Optical Coherence Tomography Studies. Neurology. 2021 Jul 13;97(2):68-79. doi: 10.1212/WNL.0000000000012125.
